# The Hepatic Axis Fructose-Methylglyoxal-AMPK: Starring or Secondary Role in Chronic Metabolic Disease?

**DOI:** 10.3390/jcm14103559

**Published:** 2025-05-19

**Authors:** Alejandro Gugliucci

**Affiliations:** Department of Research, College of Osteopathic Medicine, Touro University California, Vallejo, CA 94592, USA; alejandro.gugliucci@gmail.com

**Keywords:** fructose, DNL, dyslipidemia, AMPK, ADaM site, MASLD, methylglyoxal, glyoxalases, glycation, uric acid, KHKc

## Abstract

Biochemical alterations linked to metabolic syndrome (MetS), type 2 diabetes (T2DM), and metabolic dysfunction-associated steatotic liver disease (MASLD) may be brought on by the Western diet. Based on research conducted over the past decade, fructose is one of the main culprits. Over 80% of ingested fructose is metabolized by the liver at first pass, where it stimulates de novo lipogenesis (DNL) to drive hepatic triglyceride (TG) synthesis, which contributes to MASLD, hepatic insulin resistance (IR), and dyslipidemia. Fructose reduction produces quick and significant amelioration in these metabolic disturbances. We hereby propose potential overarching processes that can link these pathways to signaling disruption by the critical metabolic sensor AMP-activated protein kinase (AMPK). We proffer that when large amounts of fructose and glucose enter the liver, triose fluxes may be sufficient to produce transient increases in methylglyoxal (MG), allowing steady-state concentrations between its production and catabolism by glyoxalases to be high enough to modify AMPK-sensitive functional amino acid residues. These reactions would transiently interfere with AMPK activation by both AMP and aldolase. Such a sequence of events would boost the well-documented lipogenic impact of fructose. Given that MG adducts are irreversible, modified AMPK molecules would be less effective in metabolite sensing until they were replaced by synthesis. If proven, this mechanism provides another avenue of possibilities to tackle the problem of fructose in our diet. We additionally discuss potential multimodal treatments and future research avenues for this apparent hepatic AMPK malfunction.

## 1. Introduction

The Western diet may be causing biochemical changes that contribute to metabolic syndrome (MetS), type 2 diabetes (T2DM) [1,2], and metabolic dysfunction-associated steatotic liver disease (MASLD) [3,4,5]. Fructose is one chief responsible candidate since, as shown by research in the past decade: (a) its intake (especially in beverages) has much increased alongside the incidence of MetS; (b) over 80% of ingested fructose is metabolized by the liver at first pass, where it stimulates de novo lipogenesis (DNL) to drive hepatic triglyceride (TG) synthesis, which (c) contributes to MASLD, hepatic insulin resistance (IR), and dyslipidemia; (d) fructose reduction produces quick and significant amelioration in these metabolic disturbances [6,7,8,9,10].

On the other hand, research in parallel over the same period has showcased that the same processes—MetS, DNL, dyslipidemia, MASLD, and cardiovascular disease (CVD)—may occur as decreased activity of AMP-activated protein kinase (AMPK), whose physiological role would curb these anabolic processes and instead promote catabolic, energy-producing pathways [11,12,13,14,15,16].

Precisely, AMPK is a vital energy sensor in cells [17]. It detects changes in AMP:ATP and ADP:ATP ratios to maintain energy balance [18,19,20,21]. This helps maintain energy homeostasis at both the cellular and whole-body levels, making AMPK a crucial target for treating metabolic disorders. There has been, therefore, an increased interest in developing AMPK-targeted therapies, which could offer significant health benefits [14,22,23,24].

Regarding fructose, most of its metabolism occurs in the liver and begins with uncontrolled phosphorylation, which is followed by a decrease in ATP and a rise in AMP levels [25,26,27,28,29,30,31]. AMPK activation should ensue in those circumstances, decreasing lipid synthesis, boosting lipid oxidation, and decreasing glucose output.

- The opposite appears to happen, which is unexpected. Why?
- How is it that a master energy sensor that is so well conserved does not seem to react to its primary allosteric activator?
- Is it simply that the AMP disposal routes—leading to uric acid—compete effectively?
- Or rather, is there a connection to the metabolism of fructose downstream that could be hampering the due sensing by AMPK?

Indeed, fructose increases both de novo lipogenesis and glycation [27,32]. The former causes hepatic fat storage and dyslipidemia, and the latter induces carbonyl stress. Indeed, trioses from either glycolysis or fructolysis serve both as precursors of TG and methylglyoxal (MG), the most lethal glycating agent known, which, when elevated, replicates the metabolic syndrome phenotype in animal models [33,34,35].

- We question whether hepatic fat metabolism and MG are linked to these metabolic flux impairments.
- If they are indeed related, are these pathways connected at the level of an as-yet-non-characterized discrete metabolic nidus?

To provide some insight into these issues, we hereby explore the possible overarching mechanisms that could connect these pathways with disrupted AMPK signaling.

After reviewing the main features of AMPK, we address fructose hepatic metabolism, and finally, we discuss the origins and metabolism of methylglyoxal to reveal how the three biochemical pathways may be linked to the clinical trilogy of insulin resistance, dyslipidemia, and fatty liver that afflict modern society. Additionally, we suggest future research avenues for these processes, as well as potential multimodal therapies for this apparent hepatic AMPK dysfunction. We recommend that readers consult other recent in-depth studies on these subjects, as a thorough analysis of AMPK’s involvement in controlling cellular and general bodily processes, fructose metabolism, and glycation pathways is beyond the scope of this article.

## 2. AMPK a Master Metabolic Sensor

### 2.1. AMPK Senses Low Energy Status

**Figure 1:**
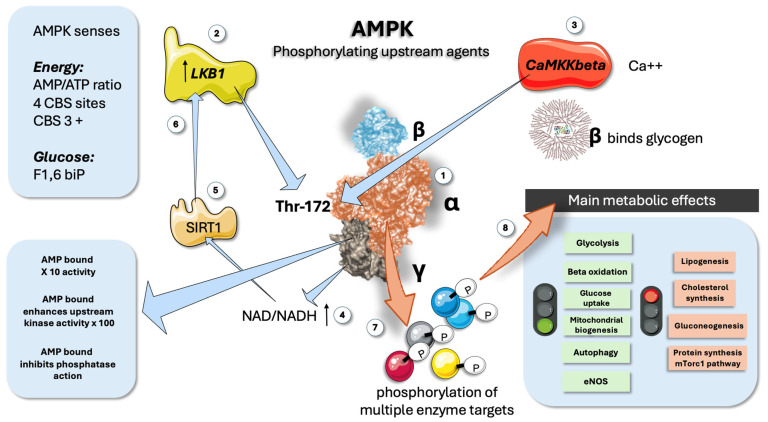
**AMPK is a key energy sensor: main features**. (1) AMPK has three subunits: alpha is catalytic, beta binds glycogen, and gamma senses AMP/ATP ratios. (2) LKB1 is one of the upstream regulators by phosphorylation of Thr172 in the alpha chain. (3) CaMKKbeta senses calcium in muscle and provides a similar upstream regulating function. (4) NAD/NADH acts as a feed-forward mechanism (5) and (6) via the participation of NAD+-dependent deacetylase SIRT1. (7) AMPK phosphorylates a plethora of downstream proteins. (8) Main metabolic consequences of AMPK activation. AMPK: AMP-activated protein kinase; LKB1: Liver kinase B1; CaMKKbeta: Calcium/calmodulin-dependent protein kinase beta; CNS: cystathionine β synthase; SIRT1: Sirtuin 1 is an NAD+-dependent deacetylase. This figure was partly generated using Servier Medical Art, provided by Servier, licensed under a Creative Commons Attribution 3.0 unported license.

AMP-activated protein kinase (AMPK) is a vital low-energy sensor in cells. It detects changes in AMP:ATP and ADP:ATP ratios to maintain energy balance. When activated, AMPK enhances ATP-generating processes and reduces ATP-consuming processes, influencing many metabolic pathways through phosphorylation of downstream targets and transcriptional regulation (Figure 1). This helps maintain energy homeostasis at both the cellular and whole-body levels, making AMPK a crucial target for treating metabolic disorders [11,12,18,21,36,37].

AMPK (as shown in Figure 1) is a heterotrimeric enzyme composed of three subunits: an alpha (α) catalytic subunit, a beta (β) scaffolding subunit, and a gamma (γ) regulatory subunit (Figure 1). These subunits are encoded by seven different genes, allowing the formation of various αβγ heterotrimers. The seven isoforms (α1, α2, β1, β2, γ1, γ2, and γ3) can theoretically combine to create up to 12 distinct heterotrimers, each with unique regulatory properties, functions, and impacts on cellular homeostasis, depending on their expression, turnover, and subcellular localization [13,15]. The α catalytic subunits feature a conventional serine/threonine kinase domain (α-KD) at the N-terminus, while the C-terminus is essential for binding to the β and γ subunits (Figure 1). For AMPK to be fully activated, the conserved threonine residue (Thr173 on the alpha 1 or Thr172 on the alpha 2 chain, respectively) must be phosphorylated. As shown in the Figure, two main upstream kinases, the tumor suppressor LKB1 and the Ca^2+^/calmodulin-activated kinase 2 (CaMKK2), have been identified [19,21,22,38]. LKB1 is constitutively active but can be further regulated through post-translational modifications. Additionally, LKB1 is a major regulator of AMPKα2, particularly in skeletal and cardiac muscle. Activation by CaMKK2 is triggered by increased cytosolic Ca^2+^ concentrations, offering an alternate pathway to activate AMPK independent of adenine nucleotide concentration changes [12,13,14]. When AMP is present, interactions between an α-linker region and the γ subunit limit its flexibility, resulting in a tighter association of the catalytic and nucleotide-binding modules, which physically protect Thr172 from dephosphorylation (Figure 1).

The β regulatory subunits have a myristoylation site at the N-terminus, which helps localize AMPK to membranes, allowing its movement to mitochondria and targeting to developing phagophores [11,13,14,15]. The β subunits also contain a central glycogen-binding domain, which modulates AMPK activity by binding to glycogen particles and co-localizing with specific targets (Figure 1). The γ subunits have a core structure composed of four tandem repeats called cystathionine β synthase (CBS) domains, which form four potential sites for nucleotide binding (Figure 1). AMP binding has a 3-proned action: 1. It potentiates AMPK 10-fold. 2. It enhances upstream kinase efficiency by 100-fold. 3. It inhibits phosphatases [15,39].

CBS1 and CBS3 serve as primary regulatory sites for AMPK activation. In the active conformation where AMP or ADP is bound, phosphorylated AMPKα-Thr172 is situated in a narrow cleft between the two modules, shielding it from dephosphorylation by phosphatases. However, only AMP binding, not ADP, can drive the enzyme’s allosteric activation. The importance of arginine residues in these sites will be highlighted later.

### 2.2. AMPK as a Druggable Target as Well as a Target for Carbonyl Stress?

AMPK signaling has attracted significant interest in recent decades due to its activation by certain pharmacological compounds. On the other side of the coin, as we shall discuss later, AMPK may be prone to modifications by unwanted metabolites. Therefore, there is significant interest in developing specific AMPK activators that directly interact with and activate the AMPK heterotrimeric complex [11,12,13,23,40,41,42].

### 2.3. The ADaM Site, a Key Allosteric Site

The interface between the beta unit glycogen binding domain and the small lobe of the alpha chain α-KD, forms a discrete pocket known as the *allosteric drug and metabolite* (ADaM) *site* (Figure 1). This ADaM site was considered for a few years, an “orphan” allosteric site since no natural AMPK ligand had been identified. Recently, acyl-Coa fatty acids have been proven to be the elusive natural modulator, suggesting an operational feed-forward mechanism [24]. Activated ADaM site seems essential for promoting AMPKα-Thr172 phosphorylation induced by agents that boost cellular AMP/ADP, making it an attractive target for pharmacological modulation of AMPK activity and a key player in our hypothesis, as we shall delve into later. This site precisely mediates salicylate action on AMPK, as well as various synthetic activators, including those that have demonstrated MASLD-improving effects [23,24,41].

### 2.4. AMPK Also Senses Glucose to Further Stimulate Its Catabolism in a Feed-Forward Mechanism That Works on the Lysosome Surface

**Figure 2:**
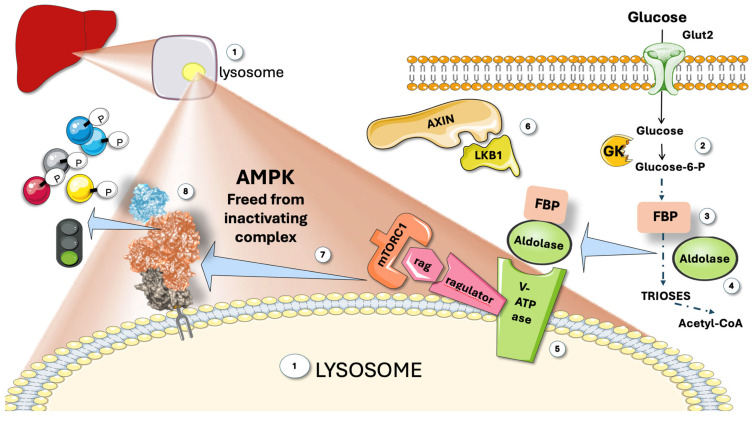
**AMPK senses glucose.** (1) Attached to the membrane of lysosomes, a complex regulating mechanism with participation of the Ragulator-Rag complex (a regulator of lysosomal signaling and trafficking) reversibly binds AMPK and inhibits it. The complex senses glucose and releases AMPK. (2) Glucose metabolism produces (3) fructose 1,6 biphosphate (FBP), which binds to aldolase (4). FBP-aldolase binds to the complex (5), releasing Axin and LKB1 (6), which frees active AMPK (7), to act on downstream proteins (8). GK: glucokinase; mTORC1; mammalian target of rapamycin complex 1; V-ATPase: Vacuolar-type ATPase (V-ATPase). This figure was partly generated using Servier Medical Art, provided by Servier, licensed under a Creative Commons Attribution 3.0 unported license.

As shown in Figure 2, aldolase, in addition to being a glycolytic enzyme, is a glucose availability sensor that controls AMPK. An AXIN-based AMPK-activation complex forms when aldolase is free of its substrate fructose-1,6-bisphosphate (FBP), whose levels sharply drop when glucose flux is low. When glucose is high, as shown in the figure, aldolase detects and binds the glycolytic intermediate fructose-1,6-bisphosphate (FBP) and attaches itself to the lysosomal surface’s v-ATPase [11,15,16,43,44]. Increased Thr172 phosphorylation and AMPK activation result from the changed interactions between aldolase and the v-ATPase in the absence of FBP. This allows the creation of an AXIN-based AMPK-activation complex that includes the v-ATPase, Ragulator, AXIN, LKB1, and AMPK (Figure 2). In addition to activating AMPK, this nutrient-sensing mechanism sets it up for future activation if the cellular energy status declines [18,20,45].

To defend cells from possible energy stress, aldolase would work as a surveillance system, detecting a drop in glucose accessible for catabolism even **before** any drop in cellular energy status had taken place. Therefore, by activating glucose-sparing oxidative pathways rather than glycolysis, this mechanism would prepare cells for future low glucose availability conditions [18,20,43,45,46].

## 3. Fructose Metabolism: Overview

**Figure 3:**
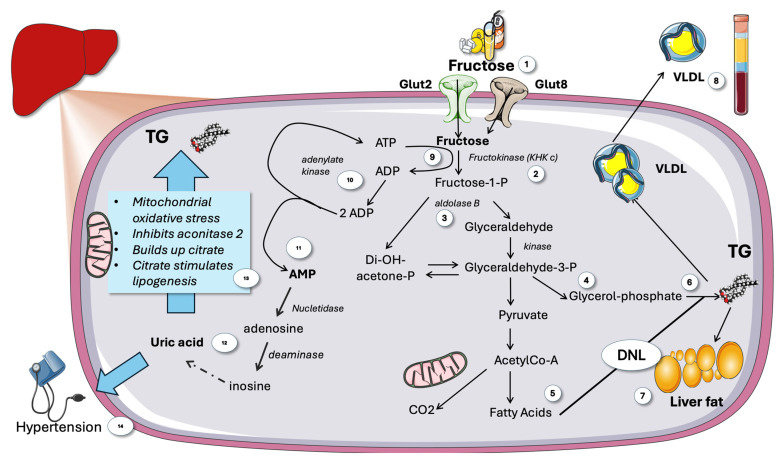
**Overview of fructose metabolism in the liver.** (1) Fructose is taken up by Glut2 and 8. (2) Fructose is phosphorylated by KHKc then split by aldolase b (3) to yield trioses (4) that can be metabolized to acetyl-Co-A and oxidized or yield fatty acids (5) in DNL, which can result in TG using trioses as backbones (6) that may accumulate as liver fat (7) or secreted as VLDL (8). Phosphorylation of fructose consumes ATP (9), lack of feedback regulation depletes cytosolic ATP leading to compensation by recycling 2 ADP by adenylate kinase (10). A transient increase in AMP ensues (11) signaling low energy status that should active AMPK. AMP is catabolized to uric acid by a series of purine enzyme catabolic enzymes (12). Uric acid stimulates lipogenesis by partially blocking the Krebs cycle (13) and hypertension (14). KHK: ketohexokinase c. VLDL: very low-density lipoprotein. TG: triglycerides. DNL de novo lipogenesis. This figure was partly generated using Servier Medical Art, provided by Servier, licensed under a Creative Commons Attribution 3.0 unported license.

The Western diet, characterized by high intakes of refined sugars, fats, and processed foods, has become prevalent in many parts of the world. This dietary pattern is typified by the consumption of table sugar and high-fructose corn syrup (HFCS) [47,48,49]. Fructose in either of these, found in sweetened beverages and food, has seen a dramatic rise in consumption over recent decades. This increase parallels the rise in MetS, T2DM, and MASLD, suggesting a possible causal relationship. Conventional wisdom has maintained for the last half century that fructose metabolism is mainly the function of the liver [27,49,50]. On the other hand, according to new and important research, at low intake amounts, intestinal fructose metabolism may shield the liver from excessive exposure to dietary fructose, where its metabolism may be more detrimental [51,52]. Fructose is absorbed through the intestinal epithelial cells’ apical membrane via an energy-independent pathway (Figure 3).

Most of the absorbed fructose enters the circulation through the basolateral membrane of enterocytes thanks to the associated GLUT2 transporter (reviewed in [29]). The intestines thus function as a barrier and may convert fructose into glucose or lactate when small amounts of sugar are eaten, as in fruit. The portal system then carries the glucose or lactate to the liver. Conversely, numerous adverse effects can arise when fructose consumption is significantly higher, as it is in many American teenagers (where it may account for up to 25% of caloric intake). The microbiota metabolizes excess fructose to create acetate, which is then sent to the liver through the portal system and participates—together with fructose itself—in the development of hepatic IR, as we shall discuss later (reviewed in [29]). Moreover, because fructose encourages the growth of undesirable microbiota, it causes leaky gut, which has detrimental inflammatory effects and lets dangerous substances enter the liver. DNL is the metabolic pathway by which fatty acids are synthesized from non-lipid precursors [49,53,54,55]. The accumulation of TG in the liver has several downstream effects that are detrimental to metabolic health. One of the primary outcomes is the development of MASLD, characterized by excessive fat buildup in liver cells. MASLD can progress to more severe forms of liver disease, including non-alcoholic steatohepatitis (NASH), fibrosis, and cirrhosis. Increased hepatic TG also contributes to hepatic insulin resistance [56,57,58,59,60,61,62,63,64,65,66]. Hepatic insulin resistance can then alter the production and secretion of lipoproteins, leading to an unfavorable lipid profile. Elevated levels of very-low-density lipoproteins (VLDL) and small-dense low-density lipoproteins (sd-LDL), coupled with decreased high-density lipoproteins (HDL), are common in individuals with MetS and MASLD. This dyslipidemia is a major risk factor for cardiovascular diseases [22,67].

### 3.1. Hepatic Fructose Metabolism Differs from Glycolysis

#### 3.1.1. KHKc Quick Unregulated Activity May Deplete Cytosol ATP

**Figure 4:**
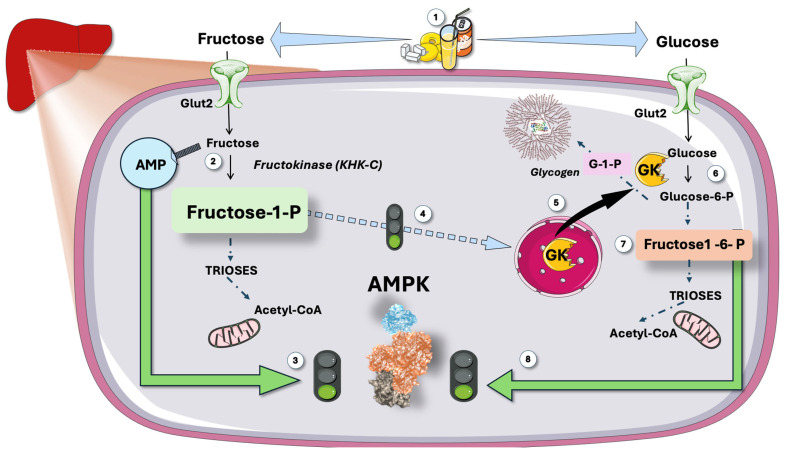
**Predicted effect of hepatic metabolism of large intake of sugar: synergic double stimulation of AMPK**. (1) large intake of sugar (i.e., some teenagers can get up to 25% of their caloric intake this way) results in (2) rapid, unregulated fructose phosphorylation with raises of AMP, which activates (3) AMPK via the CBS repeats and its metabolite fructose-1-P (4) activates GK. On the other hand, glucose is phosphorylated by this GK (6) and later yields fructose 1,6 biphosphate, which binds to aldolase and activates AMPK (8). Fructose then stimulates glucose metabolism, which further stimulates AMPK. GK: glucokinase. CBS: cystathionine β-synthetase. This figure was partly generated using Servier Medical Art, provided by Servier, licensed under a Creative Commons Attribution 3.0 unported license.

Three distinct enzymes are used in the non-tightly controlled, primarily hepatic, metabolism of fructose (Figure 4). As soon as fructose enters the cell, three enzymes in a cascade conduct fructolysis [27,28,57,65]: these are ketohexokinase c (KHKc), aldolase b, and glyceraldehyde kinase. In a nutshell, as shown in Figure 3, fructose yields triose-phosphates, which are subsequently added to the cellular pools created by glycolysis and gluconeogenesis. The transcription factor, carbohydrate-response element-binding protein (ChREBP), is activated by the consequent rise in carbohydrate metabolites, which controls hepatic de novo lipogenesis (DNL). As shown in the figure, depending on the fluxes, hormone environment, and other circumstances, different percentages of the trioses pool are converted into acetyl-coA, glycerol backbones in TG, glycogen, lactate, or fatty acids via the DNL process [27,28,68,69].

KHKc, also known as fructokinase, catalyzes the rapid and irreversible phosphorylation of fructose to fructose-1-phosphate (Fructose-1-P). This metabolite is found only in the fructolytic pathway. KHKc is not allosterically inhibited by ATP nor by its product. Moreover, the ability of KHKc to phosphorylate fructose is ten times larger than the ability of glucokinase (GK) to phosphorylate glucose [70,71,72,73].

From the standpoint of evolution, this is unquestionably a good method for processing fructose, a rare and intermittent commodity in the diet, as quickly and efficiently as possible. Clearly, it has become a liability for our current diet [68,74]. Fructose-1-P can thus rapidly accumulate to millimolar concentrations in hepatocytes. Therefore, as shown in Figure 3, fructose phosphorylation-induced reduction in ATP leads to recycling of 2 ADPs into one ATP and one AMP. This increase in AMP/ADP or AMP/ATP ratio should result in strong AMPK activation. Paradoxically, as shown later, the opposite occurs.

#### 3.1.2. Uric Acid Amplifies Fructose Damage

AMP produced by unrestricted KHKc action after large doses of fructose efficiently activates nucleotidases and AMP deaminase and subsequent uric acid production (Figure 3), more so if ingestion of fructose is high and quick (sugar-sweetened beverages; fruit juices). Uric acid itself contributes significantly to hypertension, inhibits mitochondrial activity, and encourages more fructose metabolism [5,68,75,76,77]. In humans, fructose taken intravenously and orally has been demonstrated to cause ATP depletion. Similarly, after consuming fructose, there is an abrupt increase in uric acid [5,75]. The key point here is that fructose differs from glucose in that it causes a brief drop in intracellular phosphate and ATP levels during metabolism, which is linked to AMP terminal nucleotide turnover and the consequent production of uric acid (Figure 3).

A temporary halt in protein synthesis, an increase in oxidative stress, and mitochondrial dysfunction are among the reactions brought on by this drop in ATP levels, and these events ultimately play a crucial part in fructose-mediated effects, but there is more, as we shall delve into later.

#### 3.1.3. Fructose and Lipid Metabolism

The deleterious effects of fructose on metabolism were first discovered due to its harmful effects on lipids (Figure 3). This includes the ability of very high fructose exposure to cause fasting hypertriglyceridemia and steatosis, as well as increased postprandial lipemia, after only a few days of exposure. Increased hepatic DNL (the metabolic pathway that produces new fatty acids from precursor molecules) has been related to these changes in the liver and circulating TG levels [28,54,55,69,78,79,80,81]. Carbohydrate consumption, particularly when consumed as simple sugars and in liquid form, promotes hepatic lipogenesis even when maintenance dietary interventions are implemented, according to studies that looked at the effects of higher CHO/sugar/fructose consumption [58,60,82]. Both intervention trials that increased sugar/fructose intake and those that decreased fructose intake (including ours) provide evidence that sugar/fructose intake influences hepatic DNL [10,29,69,80,81,83,84]. Indeed, KHKc deletion or inhibition are both effective at protecting against fructose-induced metabolic abnormalities. Clinical research has shown that MASLD patients have higher levels of KHKc expression in their livers and that decreasing the amount of KHKc induces a decrease in liver fat. All these data support the development of KHKc inhibitors, which are now being developed for human use [71,72,73,85,86].

#### 3.1.4. Fructose Stimulates Glycolysis

*Fructose is not ingested alone; it is usually paired with glucose in sugar or HFCS.*

Another reason why sugar ingestion should activate AMPK (but does not appear to do so) is that fructose is rarely consumed alone; instead, it is most frequently combined with glucose in sugar or high-fructose corn syrup (HFCS). As shown in Figure 4, fructose-1-phoshate (F-1-P), the first product in fructolysis, is a strong stimulator of GK; therefore, of glycolysis. Glycolysis produces F-1-6 biP, which, in turn, activates AMPK through the aldolase sensing mechanism described above and in Figure 2 [87,88,89,90,91,92].

Therefore, sugar consumption should activate *both AMPK sensing mechanisms: low ATP and concurrent glycolysis.*

## 4. The Main Paradox

In the liver, *rapid fructose and glucose metabolism leading to surges of AMP and F-1-6 bi P should strongly activate AMPK.* As recalled in Figure 4, fructose surges in the liver strongly increase AMP concentrations, for which AMPK has evolved to respond.

In lieu of AMPK becoming more active, the well-documented final action of fructose suggests that it is unresponsive. Why? One explanation is that the drive of fructose metabolism for lipogenesis promotion overrides AMPK activation, although this does not make much sense for such an exquisite sensor of AMP/ATP concentrations.

Another possible explanation is the action of uric acid. Indeed, uric acid per se acts to amplify lipogenesis, as shown in Figure 3. Fructose also induces oxidative stress in the mitochondria via uric acid, activating NADPH oxidase, which then moves to the mitochondria. Aconitase-2 (part of the Krebs cycle) and enoyl CoA hydratase (part of β-oxidation) are two of the many enzymes in the mitochondria that are known to be susceptible to oxidative stress. Fructose and uric acid reduce the activity of aconitase-2, which results in a buildup of citrate that enters the cytoplasm and initiates lipogenesis by activating ATP citrate lyase. Mitochondrial oxidative stress triggers endoplasmic reticulum (ER) stress, activation of sterol regulatory element binding protein 1c (SREBP-1c), and further stimulation of lipogenesis by activating FAS and acetyl CoA carboxylase-1 (ACC) [60,68,74,75].

*But why is all of this happening on a surge of AMP that should activate AMPK and curtail lipogenesis? Is this a flagrant failure of a so well-honed mechanism evolved over millions of years?*

Some authors suggested that there is competition for the substrate AMP between AMP deaminase (AMPD, the first enzyme in purine nucleotide catabolism, on its route to uric acid) and AMPK. AMPD would sequester most of AMP, preventing it from being sensed by AMPK [68,76]. Although not unrealistic, this contention does not seem very plausible. Some other mechanism must be operative in these conditions, as we discuss in the following section.

## 5. Fructose Metabolism Produces Trioses That Branch into TG and Methylglyoxal Synthesis

**Figure 5:**
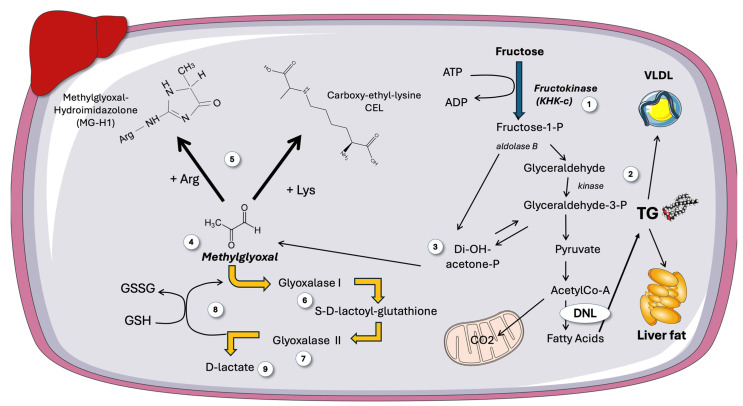
**Fructose can produce methylglyoxal, a very reactive, toxic molecule**. A more in-depth view on the destiny of trioses stemming from fructose metabolism (1), which can be the backbone of TG (2), as shown in Figure 5, or (3) decompose into methylglyoxal (MG) (4). MG is a very reactive dicarbonyl that forms deleterious adducts with Arg to form MGH-1 and Lys residues to form CEL in proteins (5), as well as with nucleotides and lipids. MG is detoxified by Glyoxalase 1 (6) and 2 (7), with consumption of reduced glutathione (8), the end product being D-lactate (9). DNL: de novo lipogenesis; GSH: reduced glutathione; MG: methylglyoxal; MGH-1: methylglyoxal-hydroimidazolone 1; CEL: carboxy-ethyl-lysine. This figure was partly generated using Servier Medical Art, provided by Servier, licensed under a Creative Commons Attribution 3.0 unported license.

As shown in Figure 5, trioses from fructose catabolism may serve as sources of glycerol for TG synthesis but also are sources of methylglyoxal [34,35,93,94,95,96,97]. Increased MG stems the from spontaneous decomposition of unstable DHAP. Therefore, increased triosephosphates (TP) are important bridges in the pathways of glycerophosphate (α-GP) synthesis, glycolysis, gluconeogenesis, and MG production [33,34,35]. Of note, then, is the fact that MG production rises in proportion to the flux of trioses needed to promote TG synthesis. The presence of several conserved enzymes involved in the [35,97] detoxification of methylglyoxal highlights its biological significance [96,97]. Indeed, when MG reacts with Arg or Lys residues in proteins, a hydrophilic, positively charged Arg or Lys residue is swapped out for an uncharged, hydrophobic residue, which causes misfolding and triggers the unfolded protein response (UPR). UPR is a driver of insulin resistance and low-grade inflammation. As depicted in Figure 5, evolutionarily conserved enzymes known as glyoxalases, including Glyoxalase 1 and 2 (Glo1 and 2), are involved in the detoxification of reactive methylglyoxal and glyoxal, which are ultimately transformed into D-lactate or glycolic acid, respectively. These enzymes are essential for preventing diseases caused by the buildup of MG-mediated alteration of amino acids or nucleotides, according to studies employing knockdown of these enzymes in mice, cell culture, and worms [32,35,97]. The end product, D-lactate (as opposed to L-lactate, the end result of glycolysis), can be detected in peripheral blood and acts as a surrogate measurement of whole-body MG synthesis. The fact that an increased, uncontrolled triose flux induced by fructose consumption might increase the synthesis of MG has not been given enough consideration [32,98,99,100].

## 6. Human Research Corroborates Increased MG Fluxes Induced by Fructose

One qualitative and indirect measure of MG flow is plasma D-lactate levels. Adult obesity has been linked to elevated levels of serum MG and D-lactate. Nevertheless, neither this phenomenon nor the connection between these pathways had been investigated in relation to childhood obesity. To shed some light on these issues, we conducted a case-control study as well as an intervention study in adolescents [98,99,100].

We compared adolescents with obesity and high fructose consumption vs. adolescents with normal weight. This cross-sectional study of obese adolescents without overt MetS, provided evidence of early proatherogenic changes in lipoprotein profiles, high prevalence of sd-LDL, and incipient structural changes in carotid arteries measured by CIMT and endothelial function. The key findings were as follows: (a) the first proof of elevated D-lactate levels, a surrogate marker of MG and, consequently, triose-phosphate fluxes, in obese adolescents; and (b) a strong correlation between D-lactate, LDL size, and sd-LDL, indicating a potential shared connection between the two derangements linked to early IR. As described earlier, from a mechanistic perspective, elevated D-lactate levels would most likely result from increased MG through fructose-induced triosephosphates (TP) fluxes [99,100]. In a second study, we wanted to determine if fructose could produce more metabolic disruption than glucose due to its increased lipogenic potential and stronger MG production stimulation [98,99]. The intervention in obese adolescents involved reducing the quantity of sugar in the diet from 28% to 10% of total calories and replacing it with the same amount of refined starch for nine days. We demonstrated improvements in insulin kinetics, a decrease in liver fat, and a decrease in hepatic DNL together with correction of dyslipidemia. We would not have anticipated a significant change in either serum D-lactate levels or DNL levels if glucose were a substrate that was equal to fructose for the formation of either MG or DNL, because the participants consumed the same amount of carbohydrates, therefore they increased the amount of glucose in their intervention diets [98,99,100].

Thus, we concluded that fructose, regardless of its caloric equivalent, contributes significantly to obesity and metabolic syndrome, in part by raising the formation of hepatic MG (and consequently its detoxified metabolite D-lactate). Increased hepatic MG flow may cause fructose-induced oxidative stress through several mechanisms, including its own catabolism [101]. For instance, as shown in Figure 5, to provide the sulfhydryl group required for the hydration of MG to generate D-lactate, the detoxifying enzyme Glo1 needs the antioxidant glutathione in its reduced form (GSH). However, a diet heavy in processed foods and sugar, like the Western diet that our participants had, reduces the pool of this antioxidant. In this regard, it must be noted that potential therapies for metabolic syndrome have been developed, including small chemical inducers of Glo1 expression [95,97].

## 7. Surges of Hepatic Fructose and Methylglyoxal May Hinder AMPK Sensing of AMP as Well as the Physiological AdaM Site Metabolite: A Synergic Double-Hit?

**Figure 6:**
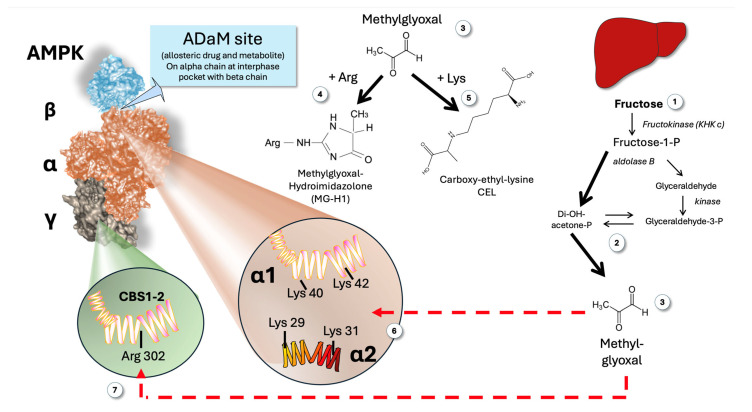
Proposed mechanism linking fructose-mediated MG production and the paradoxical apparent lack of response of AMPK in the face of hepatic low AMP produced by alimentary fructose surges. Fructose metabolism (1) produces increased triose flux (2) and MG (3) that can modify Arg residues to MGH-1 (4) and Lys residues to CEL (5). The ADaM site (interphase alpha-beta chain as shown in the Figure) alpha chain contains two critical Lys residues for its function: alpha 1 Lys 40 and Lys 42; alpha 2 Lys 29 and 31 (6). These are targets for transient increases in MG induced by fructose (3). The CBS 1 and 2 contain an Arg 302 residue that is important for optimal conformation and regulatory function (7). This is another potential target for the fructose-induced transitory increases in MG. MG: methylglyoxal; ADaM: allosteric drug and metabolite; CBS; cystathionine β-synthetase; MGH-1: methylglyoxal-hydroimidazolone 1; CEL: carboxy-ethyl-lysine.

Figure 6 illustrates a possible outcome of abrupt increases in MG brought on by the metabolism of fructose in the liver. We propose that MG may form adducts with critical arginine residues in both the CBS (AMP-sensing sites) and critical lysine residues in the ADaM site, leading to sudden (obviously transient) and potentially synergic “blinding” of AMPK.

### 7.1. Impairment of the Gamma Regulatory Subunit

As shown in Figure 6 (as well as in Figure 1), the γ subunits have a core structure made up of four tandem repeats called cystathionine β synthase (CBS) domains [13,16,43]. CBS1 and CBS3 serve as the primary regulatory sites for AMPK activation. Of note, CBS 1 and CBS 2 (although quantitatively less critical than CBS 3, they are still important) Arg 302 to be functional, as proven by site-directed mutagenesis studies [11,15]. If that Arg 302 is attacked by MG with the formation of MG hydroimidazolone-1 (MGH-1)—the main intracellular Maillard adduct—it may possibly impair AMP sensing by these CBS (Figure 6).

### 7.2. Impairment of the ADaM Site

Recent research on mutated AMPK has determined that the alpha 1 chain needs two free lysines, Lys 40 and Lys 40; (or alpha 1 chain Lys 29 and Lys 31), to be functional. Thus, it is likely that MG may also impair the ADaM site sensing of its known allosteric activator: long-chain acyl-CoA, a feedforward mechanism [11,15,16,24,40,43,44].

If these biochemically plausible mechanisms were to be proven experimentally, we would be in the presence of a synergic negative effect on a key metabolic sensor. Indeed, combination strategies involving compounds binding at these different sites resulted in a synergistic effect on AMPK phosphorylation and physiological effects in intact cells [102]. The same reasoning may be applied to the putative effects of MG at both the AdaM and the CBS 1 sites, in this case, it would be a combined and synergistic negative effect.

## 8. Recapitulating Our Argument

When important loads of sugar reach the liver, on the one hand (left in Figure 4) fructose metabolism leads to well-documented quick ATP consumption and raised AMP levels that should activate AMPK. On the other hand, glucose metabolism (cross-stimulated by parallel fructose metabolism) would lead to further AMPK activation via the F,1-6 bi P-aldolase mechanism as depicted on the right side of Figure 4. As a result, there are two distinct allosteric sites that should override the strongly lipogenic activity of fructose and uric acid.

However, current data suggest that classical anabolic pathways that should be inhibited by AMPK consistently appear to be activated by fructose (DNL, cholesterol synthesis, VLDL production, glucose output) and vice versa for catabolic ones. A possible scenario is that lipogenic effects induced by carbohydrate-responsive element-binding protein (ChREBP) stimulation, override AMPK signaling. Because it involves a core metabolic sensor that has been preserved by evolution, this option seems to defy logic. Instead, we propose another mechanism that may enhance the potent lipogenic activity of fructose, stemming from its own metabolic cascade and sharing a common intermediate that is essential for lipogenesis: DHAP (one of the trioses).

Indeed, DHAP, a very unstable compound, decomposes spontaneously into MG-a very highly reactive dicarbonyl, that attacks Arg and Lys residues in proteins (as well as nucleotides). Glyoxalases have evolved precisely to degrade it into D-lactate. We and others have shown that D-lactate is high in the presence of obesity and high fructose consumption. Moreover, our intervention studies have shown that fructose restriction is very effective in reducing it in just a few days. It is evident from this that fructose raises MG in humans, which in turn promotes its catabolism and the elimination of this molecule [35,97,98,99,100].

Structural features of CBS 1 and 2 AMPK show important Arg residues that are essential for sensing low-energy status. More recently, it has been shown that the ADaM site necessitates two free Lys residues for its allosteric metabolite binding. These sites are targets of MG adduct formation.

## 9. Towards a Multiprong Approach to Counter the Proposed Hepatic Fructose-MG-AMPK Axis?

**Figure 7:**
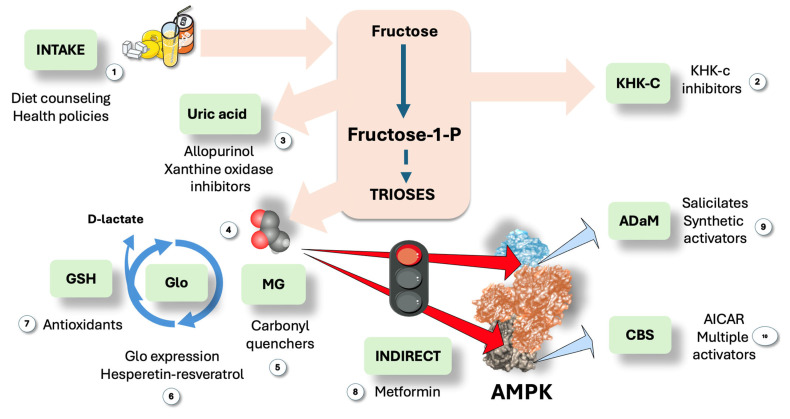
**Suggested actions to modulate the proposed pathway: room for a multi-site synergistic activation approach?** We propose that triose fluxes may be sufficient to generate short increases in methylglyoxal (MG) when large amounts of fructose and glucose enter the liver, allowing steady-state concentrations between production and Glo activity to be high enough to cause liable AMPK residue change. Therefore, the first line of action would be (1) dietary restriction through proper information and active health policies. Considering therapeutic options, the following logical step is (2) KHKc inhibitors, promising and already in clinical trials, together with (3) uric acid therapies since uric acid feedforward fructose metabolism and is deleterious per se. Further down the pathway, MG (4) could be quenched (5), and effective, not toxic drugs need to be developed. Glo1 expression can be stimulated (6) by hesperetin-resveratrol or antioxidants (7) may help recycle GS-SG. Next, AMPK can be activated via indirect action, i.e., exercise or metformin (8). Finally, direct activators may be employed on the ADaM site (9), such as salicylates and many under development; and/or the CBS sites (10) for which molecules are under study as well. MG: methylglyoxal; Glo: glyoxalases. This figure was partly generated using Servier Medical Art, provided by Servier, licensed under a Creative Commons Attribution 3.0 unported license.

The mechanism we are proposing would strengthen the need for actions to counter these processes, be it as diet modification supported by targeted health policies or through continuing drug development towards multiple targets, some of them already in clinical trials. In Figure 7, we summarize the main existing and suggested targets in this axis pathway.

### 9.1. Education, Health Policies and Fructose Restriction

If proven in future research, our contention would lend credence to a fructose restriction-focused intervention as a strategy to address IR, enhance insulin kinetics, and reduce dyslipidemia.

The effectiveness of long-term fructose restriction in preventing or treating MASLD and its related metabolic aftereffects will need to be investigated further. However, this mechanism offers another argument in favor of current initiatives to lower sugar intake to enhance metabolic health, such as that of the American Heart Association (AHA). The AHA advises limiting added sugars, including fructose, to no more than 6% of daily calories. By contrast, many American teenagers consume four times more: 25% of their calories are sugar [103]. The AHA emphasizes that excess fructose from added sugars, contributes to higher triglyceride levels, which can increase the risk of cardiovascular disease [103,104].

### 9.2. Pharmacological Approach

Several drugs are in the pipeline to increase AMPK activity as well as to reduce fructose, uric acid, and methylglyoxal effects. What about a holistic approach at multiple levels?

#### 9.2.1. AMPK Activators

As recalled in this article, numerous fascinating details on the structure, regulation, and physiological functions of AMP-activated protein kinase (AMPK) have emerged since it was identified as a key regulator of energy balance. It has been difficult to create direct activators of AMPK that produce positive effects, even while exercise, calorie restriction, metformin, and many natural items raise AMPK activation and have a variety of health advantages. Nonetheless, as shown in the figure, a few direct AMPK activators have reached clinical trials after being discovered and evaluated in preclinical models in recent years [23,41]. Despite these developments, it is still unclear whether conditions or diseases are the best candidates for therapeutic AMPK activation and whether such methods are safe in the long run. It must, however, be noted that a key molecular foundation for devising therapeutic approaches based on multi-site synergistic activation is provided by the fact that AMPK has numerous ligand-binding sites and can be controlled by activators at several sites [105,106,107,108]. Research has shown that low-dose salicylate and metformin together dramatically increase ACC phosphorylation levels and activate AMPK, which effectively lowers hepatic TG content and improves the MASLD phenotype [108]. However, neither medication alone at the same dosage demonstrated these therapeutic effects. Thus, synergistic activation of the ADaM and nucleotide-binding sites may be one of the best ways to maximize AMPK activation; nevertheless, additional clinical trials are required to confirm its safety and effectiveness.

#### 9.2.2. KHKc Inhibitors

We have previously highlighted the role KHKc in the metabolism of fructose in IR, obesity, type 2 diabetes, and MASLD. For these and related conditions, KHKc inhibition is thus an appealing treatment approach. To date, three substances that target KHK have made it into clinical trials. The most sophisticated clinical KHK inhibitor, PF-06835919, was created by Pfizer and was found to be generally safe and well-tolerated at all tested doses [70,71,72,85]. It was included in multiple phase 2 trials (ClinicalTrials.gov Identifiers: NCT05463575, NCT03969719, NCT03256526, NCT06089265). At a dose of 300 mg, PF-06835919 (1) demonstrated effectiveness in lowering liver fat in NASH patients. Eli Lilly has started Phase 1 trials for two KHK inhibitors that are being developed for diabetes and NASH (ClinicalTrials.gov Identifiers: NCT04559568, NCT04270370). Another strong zwitterionic KHKc inhibitor BI-9787 is undergoing animal trials [70,71,72,73,86].

#### 9.2.3. Methylglyoxal Quenchers and Glo1 Promoters

Despite being strong MG scavengers, molecules such aminoguanidine and phenacylthiazolium bromide were discovered to be hazardous and unstable, respectively. Increasing the expression and activity of Glo1 is a more viable and possibly successful strategy. Glo1 effectively combats MG-linked dicarbonyl stress because it catalytically metabolizes MG at diffusion-limited rates. Small-molecule activators of the transcription factor nuclear factor-erythroid factor 2-related factor 2 (Nrf2) can induce the expression of Glo1 [95,97]. Trans-resveratrol produced the highest maximal response of the Glo1-ARE (antioxidant response element) transcriptional activity, while hesperetin produced the lowest median effective concentration. Pharmacological synergism was produced by combining these two substances. The first clinical trial using the Glo1 inducer, trans-resveratrol and hesperetin combo (tRES-HESP), a randomized, double-blind, placebo-controlled crossover phase 2A research, was successfully finished. tRES-HESP reduced low-grade inflammation, fasting hyperglycemia, and insulin resistance [33,95]. Of note, resveratrol stimulates both AMPK activity and Glo1 activity: is this a useful double synergic effect?

## 10. Conclusions and Future Directions

We suggest that triose fluxes might be enough to cause brief elevations in MG when high amounts of fructose and glucose reach the liver, enabling steady-state concentrations between its generation and Glo catabolic activity to be high enough to cause AMPK residue modification. These reactions would transiently impede AMPK activation by both AMP and aldolase. These effects would boost the well-documented lipogenic impact of fructose. Given that MG adducts are irreversible, modified AMPK molecules would be less effective in metabolite sensing until they were replaced by synthesis. If proven, this mechanism provides another avenue of possibilities to tackle the problem of fructose in our diet. Indeed, the importance of fructose as a lipogenic substrate that raises liver fat and serum TG has been well documented.

Future research on protein structure, cell culture models, animal models, and fructose restriction in humans are some of the suggested avenues for demonstrating our proposed process:

1. Cell culture studies of hepatocytes exposed to high fructose and analysis of phosphorylation by AMPK of ACC (the first enzyme in lipogenesis, inactivated by AMPK by phosphorylation would offer biochemical evidence.
2. Quenching of MG by aminoguanidine using the same model would prove MG as a culprit.
3. Structural studies could follow (detection of MG-H1 and other adducts on gamma subunits or ADaM site).
4. Reduction in these effects by KHKc inhibitors and inducers of Glo1 expression would round up the evidence.
5. These findings would lend support to escalate experiments to current animal models of MetS and to reanalysis of intervention studies on fructose restriction or clinical studies on KHKc inhibition and Glo1 expression inducers.

Given the complexity of AMPK regulation, however, predicting the actual results of these potential targeted interventions is difficult. More research is required to determine whether fructose acts on AMPK directly via this proposed mechanism and the effects that are associated with it. However, this hypothesis offers yet another rationale for the need for additional research on the metabolism of fructose in humans. Therefore, it is our hope that this publication will add another field for further research to strengthen the present body of evidence that strongly supports the action taken by public health organizations to reduce sugar intake, including dietary recommendations that address “safe” limits for sugar consumption.

## Abbreviations

ACC: acetyl CoA carboxylase-1; ADaM: allosteric drug and metabolite; AICAR: 5-aminoimidazole-4-carboxamide-1-β-D-ribofuranoside; AMPD: AMP deamidase; AMPK: AMP-activated protein kinase; CaMKKbeta: Calcium/calmodulin-dependent protein kinase beta; ChREBP: carbohydrate-responsive element-binding protein; CBS: cystathionine β synthase; DHAP: dihydroxy acetone phosphate; DNL: de novo lipogenesis; FBP: fructose 1,6 biphosphate; Glo1 and 2: glyoxalase 1 and 2; GK: glucokinase; HFCS: high-fructose corn syrup; HMGcoA R: hydroxymethyl-glutaryl CoA reductase; IR: insulin resistance; KHKc: ketohexokinase c; LKB1: Liver kinase B1; MASLD: metabolic dysfunction-associated steatotic liver disease; MetS: metabolic syndrome; mTORC1: mammalian target of rapamycin complex 1; SIRT1: Sirtuin 1; SREBP-1c: sterol regulatory element binding protein 1c; T2DM: type 2 diabetes; TG: triglyceride; tRES-HESP: trans-resveratrol and hesperetin combo; UPR: unfolded protein response; V-ATPase: Vacuolar-type ATPase (V-ATPase); VLDL: very-low-density lipoproteins.
